# Enhanced Competitive Immunomagnetic Beads Assay Assisted with PAMAM-Gold Nanoparticles Multi-Enzyme Probes for Detection of Deoxynivalenol

**DOI:** 10.3390/bios13050536

**Published:** 2023-05-10

**Authors:** Kun Zeng, Jian Yang, Hao Su, Sheng Yang, Xinkai Gu, Zhen Zhang, Hongjun Zhao

**Affiliations:** 1 School of the Environment and Safety Engineering, Jiangsu University, Zhenjiang 212013, China; 2 Department of Pulmonary and Critical Care Medicine, The Quzhou Affiliated Hospital of Wenzhou Medical University, Quzhou People's Hospital, 100 Minjiang Ave, Quzhou 324000, China

**Keywords:** deoxynivalenol, gold nanoparticles, PAMAM, multi-enzyme probes

## Abstract

Contamination of deoxynivalenol (DON) in grains has attracted widespread concern. It is urgently needed to develop a highly sensitive and robust assay for DON high-throughput screening. Antibody against DON was assembled on the surface of immunomagnetic beads orientationally by the aid of Protein G. AuNPs were obtained under the scaffolding of poly(amidoamine) dendrimer (PAMAM). DON-horseradish peroxidase (HRP) was combined on the periphery of AuNPs/PAMAM by a covalent link to develop DON-HRP/AuNPs/PAMAM. Magnetic immunoassay based on DON-HRP/AuNPs/PAMAM was optimized and that based on DON-HRP/AuNPs and DON-HRP was adopted as comparison. The limits of detection (LODs) were 0.447 ng/mL, 0.127 ng/mL and 0.035 ng/mL for magnetic immunoassays based on DON-HRP, DON-HRP/Au and DON-HRP/Au/PAMAM, respectively. Magnetic immunoassay based on DON-HRP/AuNPs/PAMAM displayed higher specificity towards DON and was utilized to analyze grain samples. The recovery for the spiked DON in grain samples was 90.8–116.2% and the method presented a good correlation with UPLC/MS. It was found that the concentration of DON was in the range of ND-3.76 ng/mL. This method allows the integration of dendrimer–inorganic NPs with signal amplification properties for applications in food safety analysis.

## 1. Introduction

Mycotoxins are secondary metabolites produced by fungi, which often pollute crops in the period of growth, harvest and storage [1]. Among hundreds of mycotoxins, deoxynivalenol (DON) is isolated from corn contaminated by Fusarium graminearum and is one of the most common contaminated toxins in food crops. It was reported that 90% of the samples infected with mycotoxins contained DON [2]. DON can directly pollute crops and then influence the health of people or animals through the food chain, resulting in vomiting, anorexia, gastroenteritis, diarrhea, slow growth and even immune suppression or blood disease [3,4,5]. European Union (EU) establishes the maximum limit of DON of 750 μg/kg for cereal flour and 1750 μg/kg for unprocessed wheat [6], while the maximum limit of DON in cereals and cereal products is set as 1000 μg/kg in China [7]. To date, numerous analytical methods have been developed to detect DON in food, including high-performance liquid chromatography [8,9], liquid chromatography-tandem mass spectrometry (LC-MS) [10,11] and immunoassays [12]. Technical methods always rely on professional faculty, expensive instruments and tedious sample preparations, which would hinder their applications in the rapid screening of high-throughput samples.

Immunoassays have attracted concerns in the food safety field by the virtues of simple operations, low costs and high throughput. After specific binding between antibodies and antigens, various reporters, such as natural enzymes, fluorescein and quantum dots, have been utilized to output signals, where higher signal value will always be related to higher sensitivity. Among these reporters, enzymes are the most commonly used labels since their amazing catalytic activities would produce strong output values in a short time. To improve the performance of analytical methods further, numerous enzymes would be assembled together to form an enzyme complex to amplify the detection signals through an antigen–antibody system [13], biotin–avidin system [14] or polymers [15]. Moreover, emerging nanomaterials provide new strategies to develop an enzyme complex, which could reduce diffusion limitations and maximize the functional surface area to increase enzyme loading [16]. Gold nanoparticles (AuNPs) [17], carbon nanotubes (CNTs) [18,19] and so on have been explored. In our previous study, AuNPs and carbon nanotubes were adopted as carriers for loading horseradish peroxidase (HRP) [18]. The limit of detection (LOD) in the immunoassay based on CNTs multi-enzyme nanomaterials was improved up to 37-fold compared with conventional immunoassay, in which the higher catalytic efficiency of multi-enzyme nanomaterials played a crucial role. Although these nanoparticles served as excellent biocompatible surfaces for the immobilization of proteins, they had a strong tendency to agglomerate and non-specific binding, resulting in higher background and affecting their utility in analytical methods [20].

Dendrimers are a specific class of highly branched macromolecules with well-defined three-dimensional architecture [21]. The branching units radiate from the central monomer (i.e., core) to form the multifunctional macromolecules, in which the diameter of dendrimers increases linearly along with each generation, and the number of end groups increases exponentially [22]. Dendrimers would facilitate the controlled synthesis of inorganic nanoparticles as nanoreactors. Moreover, the high number of terminal groups at the periphery could protect the particles and increase their solubility and stability under variable conditions [23]. Furthermore, the functionalized end groups would make them the ideal host for various biological molecules, such as enzymes and antibodies. Given the structural versatility of dendrimers, the application of dendrimer–inorganic NPs has grown immensely in the field of catalysis [24], imaging [25], biomedicine [26] and environment [27]. Nevertheless, it is still less studied to apply dendrimer–inorganic NPs in immunoassay, especially for signal amplification strategies.

In this study, a competitive immunomagnetic beads assay with poly (amidoamine) dendrimer (PAMAM)—gold nanoparticles multi-enzyme probes was developed to determine the concentration of DON in food. Magnetic beads were decorated with Protein G to immobilize the antibody against DON orientationally. DON-HRP and AuNPs/PAMAM were synthesized and DON-HRP was assembled on the periphery of AuNPs/PAMAM by a covalent link. The novel magnetic immunoassay based on AuNPs/PAMAM multi-enzyme probes was established and optimized. The grain samples in local markets were collected and detected by the new method.

## 2. Materials and Methods

### 2.1. Reagents and Materials

Protein G, HAuCL4, Ovalbumin (OVA), Bovine Serum Albumin (BSA), 3,3′,5,5′-Tetramethylbenzidine (TMB), HRP, 1,1′-carbonyl-diimidazole (CDI), 1-(3-(Dimethylamino)-propyl)-3-ethylcarbodiimide hydrochloride (EDC) and N-hydroxysulfosuccinimide (NHS) were purchased from Sigma-Aldrich (St. Louis, MO, USA). The standards of DON, 3-acetyldeoxynivalenol (3-AcDON), 15-acetyldeoxynivalenol (15-AcDON), T-2 toxin, zearalenone (ZEN), ochratoxin (OTA), aflatoxin B1 (AFB1) and aflatoxin M1 (AFM1) were obtained from Beijing Zhongke Quality Inspection Biotechnology Co., Ltd., (Beijing, China). PAMAM dendrimer (ethylenediamine core, generation 4.0, amino-terminated, 5 wt% in methanol) was purchased from Weihai Chenyuan Molecular New Materials Co., Ltd. (Weihai, China). Monoclonal antibody against DON was obtained from Shenzhen Kejie Industrial Development Co., Ltd., (Shenzhen, China). Other reagents were bought from Sinopharm Chemical Reagent Beijing Co., Ltd. (Beijing, China). Immunomagnetic beads (IMBs) were purchased from Suzhou Weidu Biological Co., Ltd. (Suzhou, China).

### 2.2. Preparation of DON-HRP Colorimetric Probe

DON-HRP was developed according to the previous experiment with minor modifications [28]. About 10 mg of CDI was dissolved in 1 mL of anhydrous tetrahydrofuran (THF) and then 2 mg of DON was added. After stirring for 2 h at 25 °C, the organic solvent was removed through a vacuum pump. The products were collected and leached out in 200 μL DMSO. The mixture was added drop by drop into 1 mL of PBS containing 3 mg of HRP. After a full reaction at 4 °C for 20 h, the solution was dialyzed against 0.01 mol/L PBS at 4 °C for 48 h and the buffer was changed every 4 h. The products, DON-HRP, were stored at 4 °C until further use within two weeks.

### 2.3. Preparation of Colorimetric Probe Based on AuNPs

AuNPs with an average size of 20 nm were prepared according to the previous experiment [17]. AuNPs solution was adjusted to pH 8.0 with 0.1 mol/L K_2_CO_3_. Then 100 μL DON-HRP (1.5 mg/mL) and 1.0 mL AuNPs solution were reacted at 25 °C for 0.5 h with stirring slowly. Next, to occupy the non-specific binding site on AuNPs, 10 μL BSA (20%, *m*/*v*) was mixed with the above solution for 30 min. Finally, DON-HRP/AuNPs were gathered after centrifugation at 11,000× *g* for 15 min and then redissolved in 1 mL of PB buffer (0.01 mol/L, containing 1% PEG20000), stored at 4 °C and used within two weeks.

### 2.4. Preparation of Colorimetric Probe Based on AuNPs/PAMAM

**Figure 1:**
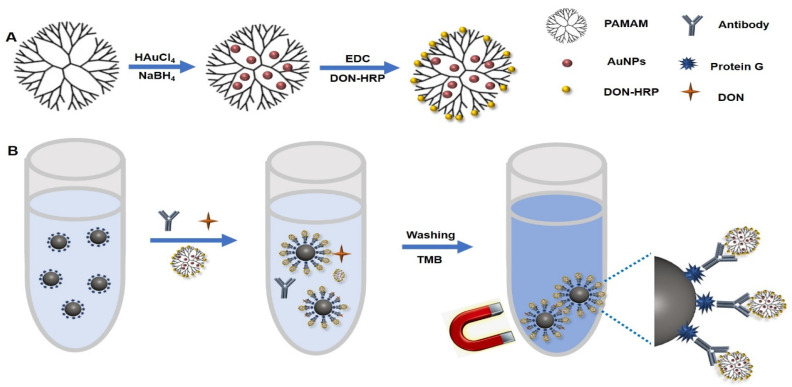
The diagram of magnetic immunoassay based on DON-HRP/Au/PAMAM: (**A**) the synthesis of DON-HRP/Au/PAMAM and (**B**) the flowchart of magnetic immunoassay based on DON-HRP/Au/PAMAM.

First, 1 mL of 30 mM HAuCL4 solution and 1.5 mL of PAMAM (1.0 wt%) were mixed for 60 min with vigorous stirring. Then 1 mL of 0.5 mol/L NaBH4 solution was added into the solution and stirred for another 30 min. The mixture was centrifuged at 12,000× *g* for 10 min to remove excess PAMAM and NaBH4 and then the precipitate, AuNPs/PAMAM, was resuspended in 1 mL ddH_2_O. AuNPs/PAMAM was mixed with 10 mg EDC and 15 mg NHS by stirring for 60 min at 4 °C and then was centrifuged at 12,000× *g* for 10 min to remove the excess EDC and NHS. Furthermore, DON-HRP was added for 12 h at 4 °C and the final product, DON-HRP/AuNPs/PAMAM, was collected by centrifugation and stored at 4 °C and then resuspended in 1 mL PBS buffer containing 1% BSA. The procedure was presented in Figure 1A.

### 2.5. Determination of Catalytic Property of Immobilized HRP

The activities of immobilized HRP in DON-HRP, DON-HRP/AuNPs and DON-HRP/AuNPs/PAMAM were explored and compared with free HRP. After protein quantification, the final HRP concentration of 0.5 ng/mL for each multi-enzyme probe was adopted to react with TMB/H_2_O_2_ mixture in a 96-well microplate. Absorbances at 652 nm were recorded every 30 s at time intervals of 0~600 s. The results were analyzed and fitted by Origin software.

### 2.6. Immobilization of Protein G on IMBs

To prepare the active IMBs, 20 mg EDC and 35 mg NHS were dissolved in 2 mL MES buffer (50 mmol/L, pH 6.0) and then 200 μL IMBs were added with continuous stirring for 4 h at 25 °C. After being collected by a magnetic separator, the active IMBs were reacted with Protein G overnight at 4 °C under gentle shaking. The products, IMBs-Protein G, were washed with MES buffer for three times and then resuspended in 1 mL PBS containing 2% skim milk and stored at 4 °C.

### 2.7. One-Step Magnetic Immunoassay with Different Signal Probes

In one plastic tube, the following reagents were added in sequence: 50 μL of IMBs-Protein G, 50 μL of mAb against DON, 50 μL of DON standards or samples and 50 μL of diluted probe (DON-HRP, DON-HRP/AuNPs and DON-HRP/AuNPs/PAMAM). After mixed fully and reacted at 37 °C for 30 min, the mixture was separated by a magnet and washed three times with 500 μL PBST. To develop the colorimetric reaction, freshly prepared TMB solution was added in drops into each tube for 20 min at 37 °C (Figure 1B). After stopping the reaction by H_2_SO_4_ (2 mol/L), the absorbance of the supernatant was recorded by a microplate reader at 450 nm.

### 2.8. Optimization of Magnetic Immunoassays with Multi-Enzyme Probes

The usage of the key elements in magnetic immunoassays was investigated. Different usage of IMBs (20, 30 and 40 μL), dilution of antibody (1:500, 1:1000 and 1:200) and dilution of multi-enzyme probes (1:50, 1:100 and 1:150) were matched with each other, and the absorbances at 0 ng/mL and 5 ng/mL of DON were recorded.

To optimize the reaction condition, solutions with different factors were utilized to prepare the analyte standards. For pH, the values were 6.5, 7.0, 7.4, 8.0 and 9.0. The concentrations of Na^+^ were 0, 0.01, 0.05, 0.1 and 0.2 mol/L, and acetonitrile (*m*/*v*) was 0, 5%, 10%, 20% and 40%. The maximum absorbances (B0) were recorded, and IC50 was the concentration at which 50% of the antibodies were bound to the analyte.

### 2.9. Samples Preparation and Analysis

Samples of rice, corn and wheat from local markets were crushed with a pulverizer and passed a 20-mesh screen. Then, 5 g of the sample powers in a 50 mL polypropylene tube was mixed with 20 mL of PBS containing 10% acetonitrile. After vigorous mixing for 10 min, the extracts were utilized for analysis by the developed method. A total of 30 natural samples were tested and compared by the new method and ultra-high-performance liquid chromatography-tandem mass spectrometry (UPLC-MS/MS).

For UPLC-MS/MS detection, we followed the procedures in previous reports [29]. First, 5 g of the fine grain sample in a 50 mL polypropylene tube was mixed with 25 mL of acetonitrile/water (84:16, *v*/*v*). The sample was extracted on a shaker for 30 min and centrifuged for 10 min at 5000× *g*. Then 2 mL of the supernatant was passed through a solid phase extraction column, the filtrate was dried by N2 and the product was dissolved in 1 mL of acetonitrile/water (1:9, *v*/*v*). The solution was injected into Agilent 1290 UPLC coupled with ABsciex 4500 MS. The gradient elution program was carried out. Each sample was evaluated three times in duplicate.

## 3. Results

### 3.1. Construction, Optimization and Characterization of Multi-Enzyme Probes

**Figure 2:**
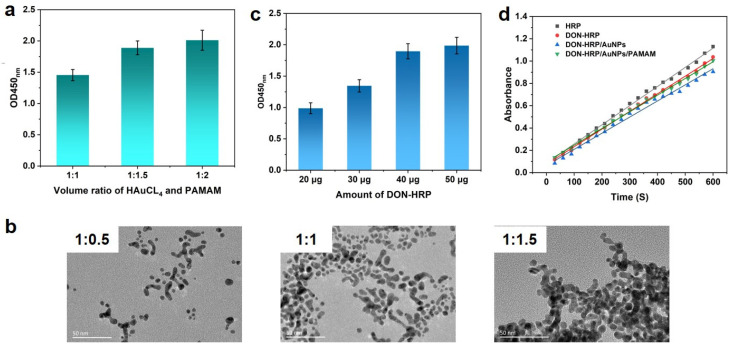
Development of multi-enzymes particles based on Au/PAMAM: (**a**) absorbance at different volume ratios of HAuCl4 and PAMAM; (**b**) TEM images of Au/PAMAM at different volume ratios; (**c**) optimization of DON-HRP; (**d**) catalytic activities for DON-HRP, DON-HRP/AuNPs and DON-HRP/AuNPs/PAMAM.

G4 PAMAM with 64 NH2 terminals was utilized as the scaffold to guest AuNPs in situ growth in this study (Figure 1A). The optimal usage of PAMAM and HAuCL4 was explored. It was observed that the absorbance changed with the volume ratio of HAuCL4 and PAMAM (1:0.5, 1:1 and 1:1.5) when 50 μg of DON-HRP was as used. The OD value at the ratio of 1:0.5 was lower than that at the ratio of 1:1 and 1:1.5 (Figure 2a). According to TEM images (Figure 2b), AuNPs/PAMAM showed an average size of 5~8 nm, smaller than conventional AuNPs (20 nm, Figure S1), and presented a dendrimer coating around metal nanoparticles. AuNPs/PAMAM at the ratio of 1:1 processed better mono-dispersity and excess PAMAM may result in the clustering of nanoparticles. Therefore, the volume ratio of HAuCL4 and PAMAM was fixed at 1:1 in the next experiment. With the help of the amino group in the periphery of PAMAM, enzyme molecules could be conjugated by covalent crosslinking to form the multi-enzymes complex. More enzymes loading on the nanoparticles carrier would contribute to the improvement of signals, which was confirmed in the results (Figure 2c). With the increasing HRP content, the absorbance improved significantly and a platform was observed over 40 μg of DON-HRP. Hence, 40 μg of DON-HRP was chosen in the next experiment.

Furthermore, the catalytic property of multi-enzyme particles was measured using TMB/H_2_O_2_ as a substrate. The concentration of HRP in multi-enzyme probes was determined through the BCA kit, and 1 μg of absolute content HRP in each multi-enzyme probe was utilized to evaluate the catalytic property. In comparison with the initial slope of catalysis kinetics, the activities of a single immobilized HRP relative to free HRP could be calculated (Figure 2d). Then, the relative activities of immobilized HRP were 91.86%, 83.72% and 88.63% for DON-HRP, DON-HRP/AuNPs and DON-HRP/AuNPs/PAMAM, respectively.

### 3.2. Optimization of IMBs Modified with Protein G

**Figure 3:**
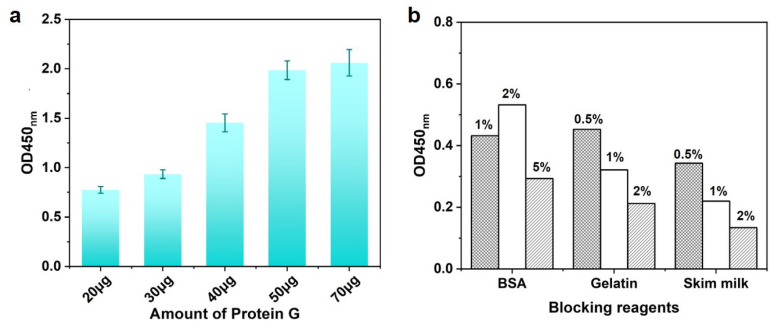
The optimization of IMB-Protein G: (**a**) optimization of usage of Protein G and (**b**) optimization of blocking reagents.

Protein G could help antigen-binding (Fab) regions of antibody orientation on the surface as far as possible (Figure 1B), which would confirm the activity of antibodies. To obtain the optimal condition, the usage of Protein G was tested when 1:1000 dilution of DON-HRP and 50 μL of IMBs were utilized. As shown in Figure 3a, the OD value increased with the concentration of Protein G below 50 μg per test, which mean the maximum loading for Protein G. To block the non-specific combination on IMBs, some reagents, including BSA, gelatin and skim milk powder, were added into the solution to reduce the background, in which 2% skim milk displayed the best performance (Figure 3b).

### 3.3. Optimization of Magnetic Immunoassay Based on Multi-Enzyme Probes

Bottomed on the reaction elements obtained above, magnetic immunoassays were established (Figure 1B). Firstly, the usage of these elements, including IMB-Protein G, an antibody against DON and multi-enzyme probes, should be explored for the best performance. Because of the competitive format, the absorbances at 0 ng/mL and 5 ng/mL of DON were recorded and the inhibition rates at 5 ng/mL of DON were calculated, in which higher absorbance at 0 ng/mL of DON and greater inhibition rates would signify the better sensitivity. According to this criterion, the optimal conditions were 40 µL of IMB-Protein G, 1:1000 dilution of antibody and 1:100 dilution of DON-HRP/AuNPs for magnetic immunoassay based on DON-HRP/AuNPs (Table S1), while 40 µL of IMB-Protein G, 1:2000 dilution of antibody and 1:50 dilution of Au/DON-HRP/PAMAM for another magnetic immunoassay (Table S2).

**Figure 4:**
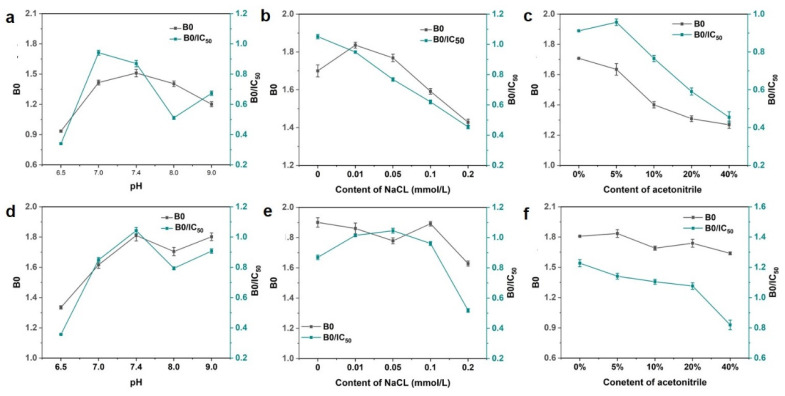
Optimal conditions in magnetic immunoassay based on DON-HRP/Au (**a**–**c**) and DON-HRP/Au/PAMAM (**d**–**f**): (**a**,**d**) optimization of pH, (**b**,**e**) optimization of ion strength and (**c**,**f**) optimization of organic solution.

Furthermore, some physicochemical factors should be evaluated. Two parameters, B0 and B0/IC50, were used to estimate the performance of these methods, in which a higher value of B0/IC50 would be associated with higher sensitivity. As shown in Figure 4a,d, it was observed that the value of B0 exhibited higher absorbance when pH exceeded 7.0 in both magnetic immunoassays. The optimal condition was at pH 7.0 and 7.4 for the two methods, respectively. The salt ion strength could interfere with the combination between antibodies and antigens but also would induce the gathering of some nanoparticles and then interfere with the characteristics of the analysis method. It was observed that the attendance of Na+ leads to the significant decreasing trend of the value of B0/IC50 in magnetic immunoassay based on DON-HRP/AuNPs, which means the loss of sensitivity. For magnetic immunoassay based on DON-HRP/AuNPs/PAMAM, the value of B0/IC50 presented minor fluctuations below 0.1 mol/L NaCl and decreased at 0.2 mol/L NaCl. Besides pH and ion strength, organic solvent needed to be evaluated since some organic solvents, such as acetonitrile, would be utilized to extract DON from food samples. The sensitivity of magnetic immunoassay based on DON-HRP/AuNPs would be influenced over 5% acetonitrile, while the higher tolerance of acetonitrile over 20% was recorded in that based on DON-HRP/AuNPs/PAMAM.

### 3.4. Analytical Performance of Magnetic Immunoassays Based on Multi-Enzyme Probes

**Figure 5:**
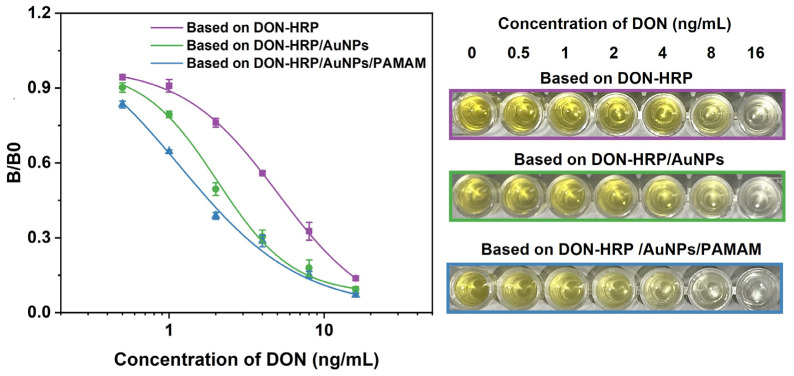
Standard curves of magnetic immunoassays based on DON-HRP, DON-HRP/AuNPs and DON-HRP/AuNPs/PAMAM.

Based on the optimal condition, magnetic immunoassays were developed with the aid of DON-HRP/AuNPs and DON-HRP/AuNPs/PAMAM, and conventional magnetic immunoassay based on DON-HRP was established as comparison, and the standard curves were shown in Figure 5. It was obvious that the two magnetic immunoassays by the aid of nanoparticles shifted to the left compared with that of conventional magnetic immunoassays. After calculation, the LODs (90% inhibition concentration) were 0.447 ng/mL, 0.127 ng/mL and 0.035 ng/mL for magnetic immunoassays based on DON-HRP, DON-HRP/Au and DON-HRP/Au/PAMAM, respectively. The linear ranges (20–80% inhibition concentration) were 1.72–12.11 ng/mL, 0.932–5.68 ng/mL and 0.13–3.12 ng/mL for the three methods. In comparison with conventional magnetic immunoassays, that based on DON-HRP/AuNPs/PAMAM showed the highest sensitivity with a 13-fold improvement of LOD, while the LOD of the method based on DON-HRP/Au was upgraded 4-fold.

### 3.5. Analysis of Grain Samples

**Figure 6:**
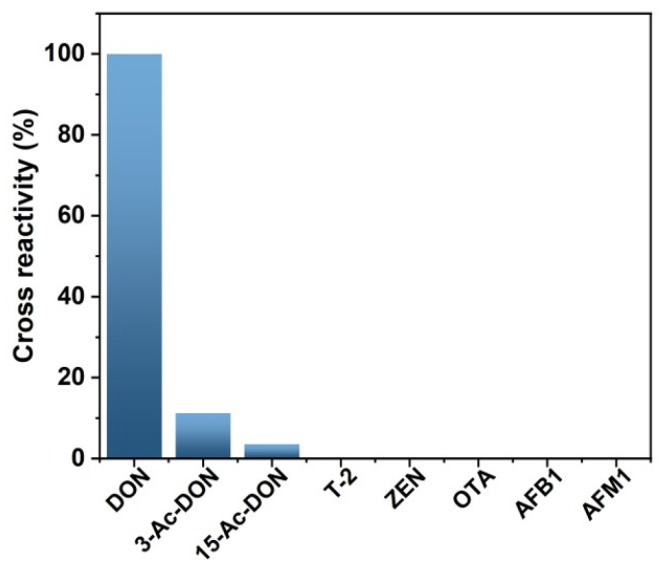
Cross-reactivity of magnetic immunoassays based on DON-HRP/AuNPs/PAMAM.

Magnetic immunoassay based on DON-HRP/AuNPs/PAMAM was utilized to determine the concentration of DON in grain samples. The selectivity of the new assay should be evaluated because of the widespread contamination of mycotoxins. The results showed that there was lower cross-reactivity with 3-Ac-DON (11.2%) and 15-Ac-DON (3.4%) and no cross-reactivity with T-2, ZEN, OTA, AFB1 and AFM1 (Figure 6). To determine the usability of the new method, rice, corn and wheat samples were spiked with various contents of DON and then tested by magnetic immunoassay based on DON-HRP/AuNPs/PAMAM. The recovery rates in rice, corn and wheat samples were 96.4–116.2%, 95.8–109.2% and 90.8–114.5%, respectively, and the coefficient variations (CVs) were in the range of 7.8–11.9% (Table 1). The higher accuracy and precision in the newly established method would guarantee reliable application in real sample analysis.

Furthermore, 30 grain samples spiked with a certain concentration of DON were determined by magnetic immunoassay based on DON-HRP/AuNPs/PAMAM and UPLC-MS/MS simultaneously. The results of the two methods presented a positive correlation (R = 0.9095) (Figure S2). Thirty samples (rice, corn and wheat) from local markets were analyzed (Table 2). The highest concentration of DON detected was 24.86 ng/mL in rice samples, 34.76 ng/mL in corn samples and 3.43 ng/mL in wheat samples, which were all below the MRL set by the EU and China. The detection rates were 40% in rice, 50% in corn and 40% in wheat samples.

## 4. Discussion

In magnetic beads assay, antibody-decorated IMBs could not only combine with antigens or analytes as solid support but also help to extract and concentrate the analytes from the sample matrix. In conventional immobilized methods, antibodies would be linked with the active group on the surface of IMBs by covalent binding or absorbance by physical reaction through hydrophobic interaction and/or electrostatic force, where their biological activity would be partially or completely lost because of the steric hindrance and the change of the conformation of the active site during fixation [30]. Antibodies with orderly arrangement would contribute to the better performance of immunoassays. Through precise design, non-functional sites in an antibody would be adopted to attach to the surface, while recognition sites could be oriented towards the solution to ensure the sufficient exposure of functional groups. Numerous strategies had been adopted to improve antibody binding, including surface modification [31], covalent attachment [32], biotin–streptavidin interaction, DNA-directed immobilization [33] and Protein G. In this study, Protein G, derived from bacteria, was introduced as a bridge to promote the orderly arrangement of antibodies since Protein G has the ability to bind the Fc fragment of antibodies specifically [34,35].

Signal amplification strategies based on nanoparticles have attracted more attention in analysis methods. Due to the high biocompatibility and large specific surface area, AuNPs serve as excellent carriers for the immobilization of proteins. However, AuNPs always tend to aggregate while the protein binding on the surface of AuNPs will be easy to form steric hindrance, which could interfere with the combination of more enzyme molecules. Dendrimers have emerged as optimal surface capping agents given their unique chemistry and structure for the controlled synthesis of metal nanoparticles [22]. According to the structure, dendrimers with a smaller generation (i.e., 0.5–2.5 generation) are usually more flexible, while higher-generation dendrimers (i.e., 3.0–5.0 generation) tend to exhibit a denser and more globular shape. It was observed that higher-generation dendrimers have been extensively applied for in situ growth of metal NPs, due to the void spaces in their architecture [36]. Furthermore, hydroxyl-, aldehyde- and amino-terminated PAMAM presented diverse capacities to host metal nanoparticles, while amino-terminated PAMAM showed higher interaction with gold nanoclusters in comparison with hydroxyl- and aldehyde-terminated PAMAM [37]. Therefore, G4 PAMAM with NH_2_ terminals was utilized as the scaffold for guest AuNPs in this study.

With the help of the amino group, more enzymes or other signal molecules could be linked with the nanostructure. Hu et al. found that electrochemiluminescence (ECL) based on PAMAM-Au-CdSe quantum dot bioprobes displayed satisfied sensitivity with the LOD of 3.7 × 10^−6^ ng/mL for the detection of brombuterol [38]. Moreover, it was found that AuNPs/PAMAM could be used as drugs or gene carriers by attaching various polymers or antibodies, which implicated that the higher molecule activity would be reserved by this nanostructure [25,26]. Similarly, the higher catalytic activities for DON-HRP/AuNPs/PAMAM were recorded compared with that of DON-HRP/AuNPs because DON-HRP/AuNPs/PAMAM presented higher relative catalytic activities. It was speculated that the outside numerous branches could provide the soft linker to combine with protein molecules and reduce spatial potential resistance, which contributed to the higher activities of the protein.

Through exploring the effect of physicochemical factors on nanoparticles in immunoassay, it was observed that AuNPs coating with PAMAM displayed higher tolerance towards high salt concentration and organic solution, compared with naked AuNPs, and further guaranteed the performance of the assays. Then we could confirm that dendrimers could assist nanoparticles to fight against the adverse factors in the environment, which would enhance the application of various nanoparticles in diagnosis, imaging, drug delivery and other fields.

After integrated with IMBs and DON-HRP/AuNPs/PAMAM, a novel competitive immunoassay was established, in which the LOD was improved 4-fold in comparison with that based on DON-HRP/AuNPs and 13-fold with that based on DON-HRP. It was suggested that multi-enzyme probes based on nanoparticles could promote the sensitivity of analytical methods with the aid of the higher specific surface area for the loading amount of HRP [18]. Next, the sensitivity of magnetic immunoassays based on DON-HRP/AuNPs/PAMAM was higher than that based on DON-HRP/AuNPs, which could be associated with the higher relative catalytic activity. In summary, dendrimer–inorganic NPs not only presented better stability but also could be undertaken as a friendly carrier to load bioactive macromolecules to play a major role in biomedicine, imaging and biosensors fields.

Various biosensors based on nanoparticles for the detection of DON have been reported in Table 3. Conventional immunosensors, such as lateral flow immunoassays or electrochemical immunoassays, were integrated with different nanostructures to establish the analysis methods. In electrochemical immunoassays, nanoparticles were adopted to modify the electrode, including hierarchical nanostructured CoCo Prussian blue analog entrapped by a Zr-based porphyrin MOF [39], multi-walled carbon nanotubes (MWCNTs) [40] and PtPd nanoparticles compound polyethyleneimine-functionalized reduced graphene oxide (PtPd NPs/PEIrGO) [41]. For lateral flow immunoassays, nanomaterials with different physicochemical properties served as signal tags, such as amorphous carbon nanoparticles (ACNPs) with strong dark color [42], fluorescent microsphere [43] and α-Fe_2_O_3_ nanocubes (FNCs) with orange color [44]. Along with the application of nanoparticles, these biosensors displayed higher sensitivity and the lowest detection limit was 0.14 fg/mL [39], implying the important role of nanomaterials in assays. Moreover, lateral flow immunoassays represented more advantages in simplicity, but the sensitivity needs to be improved. Electrochemical immunoassays displayed the highest sensitivity along with complex operation. In comparison, magnetic immunoassays based on DON-HRP/Au/PAMAM presented better performance than the majority of the listed assays [40,41,42,43,44,45].

It is important to evaluate the specificity of the immunosensors since various pollutants co-exist in real samples. The analogies displayed no obvious effects of interferences in the new method, including T-2, ZEN, OTA, AFB1 and AFM1. As biosynthetic precursors of DON, there was lower cross-reactivity with 3-Ac-DON and 15-Ac-DON because of the similar molecule structures. The results indicated that magnetic immunoassays based on DON-HRP/Au/PAMAM possessed good selectivity and specificity for DON detection.

## 5. Conclusions

In this study, AuNPs using PAMAM as a host were synthesized and characterized. Multiple enzymatic labels based on Au/PAMAM were developed and characterized. Antibodies were immobilized on IMBs through Protein G directionally. Magnetic immunoassays based on different enzyme complexes were established and compared. Magnetic immunoassays based on DON-HRP/Au/PAMAM presented the highest sensitivity with the LOD of 0.035 ng/mL, improving 13-fold compared with conventional magnetic immunoassays. The recovery of this new method for rice, corn and wheat was 90.8–116.2%. In real grain samples, the concentration of DON was ND-34.76 ng/g and the average detection rate was 43.33%. It was inspired that dendrimer–nanoparticles can yield novel strategies for universal amplification labels in biosensing and molecular diagnostics.

## Figures and Tables

**Table 1:** Recovery and precision of the established method (*n* = 4).

	Spiked Concentration (ng/mL)	Found Concentration (ng/mL)	Recovery Rate	CV (%)
Rice	0	<LOD	-	-
	1	1.16 ± 0.09	116.2%	7.8%
	5	4.82 ± 0.53	96.4%	10.9%
	10	11.21 ± 1.29	112.1%	11.5%
Corn	0	<LOD	-	-
	1	1.09 ± 0.13	109.2%	11.9%
	5	5.43 ± 0.63	108.6%	11.6%
	10	9.58 ± 1.01	95.8%	10.5%
Wheat	0	<LOD	-	-
	1	1.06 ± 0.11	106.2%	10.4%
	5	4.54 ± 0.41	90.8%	9.0%
	10	11.45 ± 1.25	114.5%	10.9%

**Table 2:** Measurement of DON in grain samples.

Sample Number	Results of Rice Samples (ng/mL)		Results of Corn Samples (ng/mL)		Results of Wheat Samples (ng/mL)	
	By the Established Method	By UPLC-MS/MS	By the Established Method	By UPLC-MS/MS	By the Established Method	By UPLC-MS/MS
1	ND ^1^	ND	8.87	4.65	2.12	1.78
2	4.56	3.43	ND	ND	ND	ND
3	ND	ND	6.86	ND	ND	ND
4	12.98	17.87	15.76	13.34	ND	ND
5	24.86	29.86	14.76	10.89	3.43	4.65
6	ND	ND	ND	ND	ND	ND
7	ND	ND	ND	ND	3.12	4.76
8	ND	ND	34.76	35.67	ND	ND
9	ND	ND	ND	ND	6.87	5.24
10	3.24	1.68	ND	1.43	ND	ND

^1^ not detected.

**Table 3:** Nanoparticles-based biosensors for the detection of DON.

**Nanoparticles**	**Biosensors**	**Recognition Molecule**	**LOD**	**Refs.**
CoCoPBA@PCN-221 nanostructure	Electrochemical immunoassays	Antibody	0.14 fg/mL	[39]
MWCNTs	Electrochemical immunoassays	Molecularly imprinted poly(l-arginine)	0.07 µM	[40]
PtPd NPs/PEI-rGO	Electrochemical immunoassays	Aptamers	6.9 ng/mL	[41]
ACNPs	Lateral flow immunoassays	Antibody	20 ng/g	[42]
Fluorescent microsphere	Lateral flow immunoassays	Antibody	2.5 ng/mL	[43]
FNCs	Lateral flow immunoassays	Antibody	0.18 ng/mL	[44]
AuNPs	Lateral flow immunoassays	Antibody	12.5 ng/mL	[45]
Fe_3_O_4_@polydopamine	Microchannel resistance sensor	Antibody	20.7 pg/mL	[46]
AuNPs	Colorimetric biosensor	Antibody	0.127 ng/mL	This work
AuNPs/PAMAM	Colorimetric biosensor	Antibody	0.035 ng/mL	This work

## Data Availability

The data that support the findings of this study are available upon reasonable request.

## Supplementary Materials

The following supporting information can be downloaded at https://www.mdpi.com/article/10.3390/bios13050536/s1, Figure S1: TEM images of AuNPs; Figure S2: Correlation analysis between magnetic immunoassay based on DON-HRP/AuNPs/PAMAM and UPLC-MS/MS; Table S1: Optimization of parameters in magnetic immunoassays based on DON-HRP/AuNPs; Table S2: Optimization of parameters in magnetic immunoassays based on DON-HRP/AuNPs/PAMAM.
