# Supplementary material for: Enhanced Competitive Immunomagnetic Beads Assay Assisted with PAMAM-Gold Nanoparticles Multi-Enzyme Probes for Detection of Deoxynivalenol
Source: Biosensors (Basel). 2023 May 10;13(5):536. doi: 10.3390/bios13050536 (PMC10216259; doi:10.3390/bios13050536)
Supplement: Supplementary file 1 [file biosensors-13-00536-s001.zip › biosensors-2289630-supplementary.pdf]

Enhanced Competitive Immunomagnetic Beads Assay Assisted with PAMAM-Gold Nanoparticles Multi-Enzyme Probes for Detection of Deoxynivalenol

Kun Zeng [1], Jian Yang [1], Hao Su [1], Sheng Yang [1], Xinkai Gu [1], Zhen Zhang [1] and Hongjun Zhao [2,*]

[1] School of the Environment and Safety Engineering, Jiangsu University, Jiangsu 212013, China
[2] The Quzhou Affiliated Hospital of Wenzhon Medical University, Quzhou People's Hospital, Quzhou 324000, China
* Correspondence: zhaohongjun@wmu.edu.cn, Tel.: +86-570-3121509

Table S1. Optimization of parameters in magnetic immunoassays based on DON-HRP/AuNPs.

		**Absorbance at 0 ng/mL of DON**	**Absorbance at 5 ng/mL of DON**	**Inhibition rate**
Volume of IMBs in one test	20 μL	1.547	0.454	29.35%
	30 μL	1.871	0.445	23.78%
	40 μL	1.934	0.460	18.61%
Dilution of antibody	1:500	1.895	0.358	18.89%
	1:1000	1.758	0.294	16.72%
	1:2000	1.354	0.245	18.09%
Dilution of DON-HRP/AuNPs	1:50	2.214	0.567	25.61%
	1:100	1.892	0.345	18.23%
	1:150	1.425	0.312	21.89%

Table S2. Optimization of parameters in magnetic immunoassays based on DON-HRP/AuNPs/PA-MAM.

		**Absorbance at 0 ng/mL of DON**	**Absorbance at 5 ng/mL of DON**	**Inhibition rate**
Volume of IMBs in one test	20 μL	1.476	0.428	29.00%
	30 μL	1.939	0.421	21.69%
	40 μL	2.010	0.349	17.36%
Dilution of antibody	1:500	1.966	0.347	17.66%
	1:1000	1.813	0.313	17.29%
	1:2000	1.360	0.222	16.33%
Dilution of DON-HRP/AuNPs/PA-MAM	1:50	2.323	0.323	13.90%
	1:100	1.963	0.276	14.06%
	1:150	1.440	0.268	18.61%

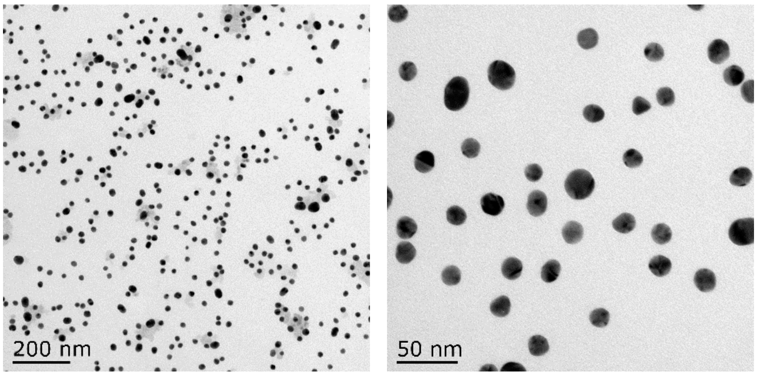

Figure S1. TEM images of AuNPs.

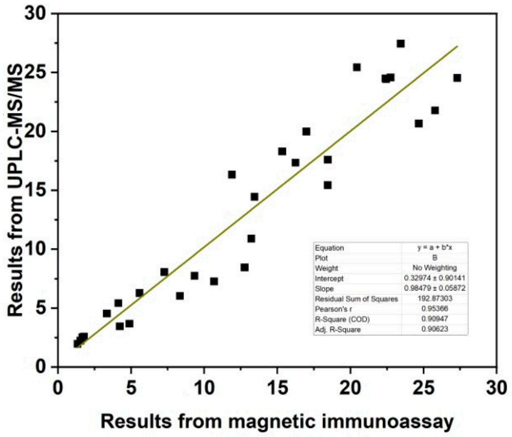

Figure S2. Correlation analysis between magnetic immunoassay based on DON-HRP/AuNPs/PAMAM and UPLC-MS/MS.
